# ATM inhibition increases the anti-tumor efficacy of radium-223 (Ra-223) against prostate cancer bone metastasis in preclinical models

**DOI:** 10.1093/jbmrpl/ziaf129

**Published:** 2025-08-05

**Authors:** Diane V Lefley, Callum G Jones, Jiabao Zhou, Helen E Bryant, Spencer J Collis, Jennifer M Down, Janet E Brown, Penelope D Ottewell

**Affiliations:** Division of Clinical Medicine, School of Medicine and population Health, University of Sheffield, Beech Hill Road, Sheffield, S102RX, United Kingdom; Division of Clinical Medicine, School of Medicine and population Health, University of Sheffield, Beech Hill Road, Sheffield, S102RX, United Kingdom; Division of Clinical Medicine, School of Medicine and population Health, University of Sheffield, Beech Hill Road, Sheffield, S102RX, United Kingdom; Division of Clinical Medicine, School of Medicine and population Health, University of Sheffield, Beech Hill Road, Sheffield, S102RX, United Kingdom; Division of Clinical Medicine, School of Medicine and population Health, University of Sheffield, Beech Hill Road, Sheffield, S102RX, United Kingdom; Division of Clinical Medicine, School of Medicine and population Health, University of Sheffield, Beech Hill Road, Sheffield, S102RX, United Kingdom; Division of Clinical Medicine, School of Medicine and population Health, University of Sheffield, Beech Hill Road, Sheffield, S102RX, United Kingdom; Mellanby Centre for Musculoskeletal Research, University of Sheffiled, Western Bank, Sheffield, S10 2TN, United Kingdom; Sheffield Teaching Hospitals HNS Foundation Trust, Weston Park Cancer Centre, Whitham Road, Sheffield S10 2SJ, United Kingdom; Division of Clinical Medicine, School of Medicine and population Health, University of Sheffield, Beech Hill Road, Sheffield, S102RX, United Kingdom; Mellanby Centre for Musculoskeletal Research, University of Sheffiled, Western Bank, Sheffield, S10 2TN, United Kingdom

**Keywords:** prostate cancer, bone metastasis, ATM inhibition, Ra-223, radiosensitization

## Abstract

Prostate cancer bone metastases are commonly treated with radium-223 (Ra-223); however, patients ultimately experience relapse. These metastases are currently incurable and there is an unmet need to improve the efficacy of Ra-223 treatment regimens. Ra-223 causes DNA strand breaks within tumor cells that are in close proximity to bone. We hypothesized that relapse is partly due to Ra-223–induced activation of DNA damage-response pathways; therefore, inhibiting DNA repair pathways with ATM inhibitors (ATMi), currently in clinical trials for other cancers, would radiosensitize bone metastases, increasing anti-tumor efficacy of Ra-223. To test this hypothesis, 2 mouse models of prostate cancer bone metastasis were administered 20 mg/kg/d ATMi on day 2, 7 or 10 and Ra-223 treatment (50 kBq/kg/wk or 300 kBq/kg/wk) commenced 24 h after first ATMi treatment. The 300 kBq/kg Ra-223 reduced PC3 prostate cancer bone metastases by 60.9%-87.4% compared with placebo. Radiosensitization with either the ATMi AZD0156 or AZD1390 prior to 300 kBq/kg Ra-223 treatment further reduced bone metastasis by 94.1% and 88.7%, respectively, whereas combining AZD1390 with 50 kBq/kg synergistically reduced tumor size and the number of mice with tumors in bone by 50% compared with Ra-223 alone. Treating early-stage RM1 prostate cancer bone metastasis with a combination of AZD1390 and 50 kBq/kg Ra-223 had no additional benefits compared with Ra-223 alone. However, delaying treatment to mimic overt bone metastases resulted in a 50% reduction in bone metastasis when ATMi was added prior to Ra-223 compared with Ra-223 alone. Notably, adding ATMi prior to Ra-223 did not exacerbate Ra-223–induced adverse effects on the bone. Instead, this treatment combination increased subsets of anti-tumor immune cells. Taken together, our data suggest that ATMi may be an effective radiosensitizer for increasing efficacy of Ra-223 in prostate cancer bone metastases, reducing Ra-223–induced adverse effects on bone.

## Graphical Abstract

**Graphical Abstract:**
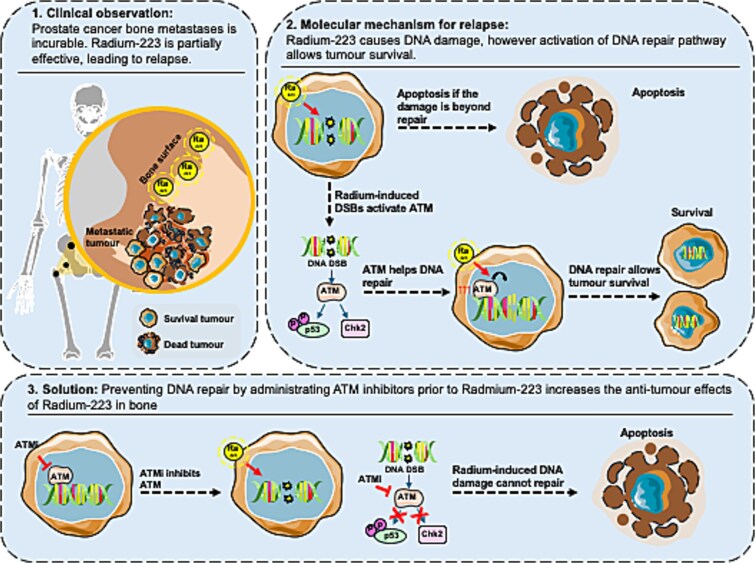

## Introduction

Prostate cancer is the second most commonly diagnosed cancer and the fifth leading cause of cancer deaths among men worldwide, with an estimated 1 467 854 new cancer cases and 379 430 deaths in 2022.^1^ The majority of deaths from prostate cancer are a consequence of tumors spreading from the prostate into other organs (metastasis), followed by building up resistance to treatments—in particular, androgen deprivation therapies (ADTs). In patients with metastatic prostate cancer, bone remains the most frequent site of metastatic disease, affecting over 80% of patients. This has serious consequences, reducing survival and the patient’s quality of life through skeletal morbidity including bone pain, fracture, and spinal cord compression. For those patients with small-volume bone metastasis, median survival is now greater than 5 yr, but patients with more extensive bone metastasis only live an average of 3 yr following diagnosis of bone involvement.^2^

In patients with metastatic prostate cancer, ADT is usually given continuously via orchidectomy or administration of anti-androgens; however, agents such as docetaxel chemotherapy and androgen receptor (AR) inhibitors such as darolutamide are being given alongside ADT earlier in the pathway when patients have hormone-sensitive metastatic disease. Indeed, in fitter patients, triple therapy has recently been introduced with ADT, darolutamide, and docetaxel given simultaneously,^3^ with significant improvement in response rates. This means that many of the current, more potent agents have already been used and, for patients who then become castrate resistant, there is therefore an urgent need for new treatments to further improve survival.^4^^,^^5^

Radium-223 (Ra-223) is a targeted alpha-emitting radiopharmaceutical, administered systemically, with a half-life of 11 d. The radiobiological effects of this drug are mainly due to the release of alpha particles with high linear energy transfer (LET) (80 keV/μm) and a very short range (<100 μm) leading to complex clustered DNA damage, including double-strand breaks.^6^ High LET irradiation is also thought to lead to oxygen-independent cytotoxic effects; this is particularly relevant in relatively hypoxic bone metastases. Radium-223 is a bone-seeking calcium mimetic; therefore, it binds preferentially to newly formed bone stroma, especially within the microenvironment of osteoblastic/osteosclerotic metastases. Because it is targeted to bone and because it releases, short-range alpha particles, Ra-223 has minimal side effects outside the target organ. It interacts with tumor cells in bone by introducing mainly double-strand DNA breaks that result in a potent and highly localized cytotoxicity. Following positive data from the ALpharadin in SYMPtomatic Prostate CAncer (ALSYMPCA) trial^7^ in 921 patients with symptomatic bone metastases and castrate-resistant prostate cancer (CRPC), where Ra-223 was demonstrated to improve survival from 11.3 to 14.9 mo (HR, 0.70; 95% CI, 0.58 to 0.83; *p* < .001), Ra-223 has been widely approved as a standard of care for men with CRPC and symptomatic bone metastasis. Radium-223 was also found to have a favorable safety profile, with minimal myelotoxicity.^8^^,^^9^

In primary androgen therapy–responsive prostate cancer, AR signaling increases the expression of DNA repair genes and, in parallel, promotes localized prostate cancer radio-resistance by accelerating repair after ionizing radiation. Indeed, ADT in combination with radiation therapy has been shown to improve cause-specific survival in men with locally advanced prostate cancer.^10^ In metastatic CRPC, studies have identified genomic defects in DNA repair genes in 20%-30% of cases, a proportion of which are germline aberrations and heritable. In addition, levels of genomic instability are high in almost all CRPC metastases. On the other hand, many CRPCs maintain high levels of AR signaling via bypass pathways or adaptations to the AR pathway,^11^ and thus the functionality of DNA repair is likely to be increased, providing a clear rationale for targeting components of the DNA repair pathway in combination with radiation. As such, there is increasing evidence of the value, in prostate cancer, of agents that inhibit DNA repair and the Poly (ADP-ribose) polymerase (PARP) inhibitor Lynparza (AstraZeneca and Merck) is now approved as standard of care in men who have a known BRCA1 or BRCA2 mutation and who have stopped responding to hormone therapy.^12^

It has been shown previously that alpha particles and other sources of high-LET irradiation activate the DNA damage response (DDR) kinases ataxia-telangiectasia mutated serine/threonine kinase (ATM)^13–15^ and ataxia-telangiectasia and Rad3-related protein kinase (ATR).^16^ In addition, AT cells (cells from patients defective in ATM) are highly sensitive to high LET irradiation^17–19^ and inhibitors of ATM increase sensitivity to high-LET irradiation including alpha particles.^14^^,^^20^^,^^21^ We, therefore, hypothesized that ATM would be an effective targeting strategy for increasing the efficacy of Ra-223 therapy for the treatment of prostate cancer bone metastases. In our current study we have therefore tested 2 orally available, potent and selective, clinically tested, ATM inhibitors (ATMi)—AZD0156 and its analog AZD1390—on their ability to increase the efficacy of Ra-223 in a BALB/c nude mouse model of human prostate cancer bone metastasis in addition to an immune-competent C57BL/6 model of prostate cancer bone metastasis.

## Materials and methods

### Cell lines

Human PC3 cells were originally purchased from the European Collection of Authenticated Cell Cultures (ECACC) and transfected to stably express Luc-2, as previously published (PC3-Luc2).^22^ Mouse bone homed RM1-BT prostate cancer cells expressing Luc-2 (RM1-Luc2) were a kind gift from Dr Ning Wang (University of Sheffield). Cells were maintained in DMEM +10% FBS (Gibco, Invitrogen, Paisley, UK) in a humidified incubator under 5% CO_2_ and used at low passage >30. Cell lines purchased from commercial sources were authenticated in-house using short tandem repeat analysis of 10 loci.

### Animals

All experiments were carried out in 6- to 8-wk-old male BALB/c nude or C57BL/6 J mice purchased from Charles River, Kent, UK. Mice were maintained in groups of 5, on a 12-h/12-h light/dark cycle, under pathogen-free conditions. Mice had free access to food (Global Diet 2018) and water. Experiments were carried out in accordance with local guidelines and with UK Home Office approval under project license P99922A2E.

### In vivo experimental procedures

To determine the effects of combining ATMi with Ra-223 on prostate cancer bone metastasis using a human-specific prostate cancer cell line, BALB/c nude mice were injected with 1 × 10^5^ PC3-Luc2 cells via left cardiac ventricle (*n* = 10/group). Seven days after tumor cell injection, mice were randomized to groups of equal tumor burden before being administered 20 mg/kg ATMi (AZD0156 or AZD1390; supplied by AstraZeneca) daily via oral gavage or subcutaneous injection (as previously published^23^). Twenty-four hours after the first ATMi administration, mice received 300 kBq/kg or 50 kBq/kg Ra-223 once per week via intravenous injection and mice were culled 25 d after initial injection of tumor cells.

The effects of combining ATMi with Ra-223 on early- and late-stage bone metastasis were determined in C57BL/6 J mice in which metastasis had been induced following intracardiac injection of 1 × 10^5^ bone-homing RM1-Luc2 cells. For early bone metastasis experiments, 20 mg/kg AZD1390 was administered daily 2 d following tumor cell injection (*n* = 10/group); for late-stage bone metastasis experiments, AZD1390 was administered daily starting 7 d or 10 d following tumor cell injection (*n* = 5/group). Twenty-four hours after injection of ATMi, mice received 50 kBq/kg Ra-223 via intravenous injection once per week and culled 14 d after initial tumor cell injection. Metastasis was measured weekly, 24 h before cull, and postmortem by IVIS Spectrum imaging 2 min following s.c. administration of 30 mg/kg D luciferin (Perkin Elmer, UK) for live animals, or 1 min following immersion in D luciferin for organs postmortem. Images from each batch were normalized and tumor volume analyzed as total photons per second. Images from dissected hind limbs were normalized and analyzed for numbers of visible tumors. All experiments were carried out in accordance with the ARRIVE guidelines, and investigators were blinded to groups of mice before analysis to remove unconscious bias in the data. On termination of experiments, hind limbs, spleens, whole blood, and serum were resected and processed for μCT, histology, ELISA, immune cell, and protein expression analysis.

### Micro-CT imaging

Micro-CT analysis of tibias from mice injected with tumor cells where detectable metastases did not form (non–tumor-bearing tibias) were carried out using a Skyscan 1172 X-ray-computed μCT scanner equipped with an X-ray tube (voltage 49 kV; current 200 μA) and a 0.5-mm aluminum filter. Pixel size was set to 4.3 μM and scanning initiated from the top of the proximal tibias. For analysis, regions of interest were drawn 0.2 mm from the tibia growth plate and analyzed for a further 0.5 mm, as previously described.^24^

### Bone histology

Non–tumor-bearing tibias were fixed in 4% paraformaldehyde (PFA) for 48 h followed by decalcification in 0.5 M EDTA for 14 d before processing and wax embedding. Three-micrometer sections were stained with tartrate-resistant acid phosphatase (TRAP) for osteoclast detection and osteoblasts were identified as mononuclear, cuboidal cells residing in chains along the bone surface, as previously described.^25^ Tumor size was taken from an average of 3 sections cut 30 μM apart. Slides were scanned on a panoramic slide scanner (3D Histech Panoramic 250) at 20× magnification and quantified in QPath. For bone cell quantification, 10× magnification snapshots were taken in QPath of the trabecular region and bone, osteoclasts, and osteoblasts highlighted using Adobe Photoshop. This image was run through TrapHisto software, as previously published.^26^

### Western blotting

Tumor-bearing femurs were snap-frozen in liquid nitrogen following dissection and stored at −80 °C prior to protein extraction using the mammalian cell lysis kit (MCL-1; Sigma, Missouri, USA). Forty micrograms of total protein was separated on a Bio-rad precast 4%-15% gel, then transferred onto polyvinylidene difluoride (PVDF) membrane (Pierce, ThermoFisher, Loughborough, UK). Nonspecific binding was blocked in 1× casein solution (Sigma-Aldrich, Missouri, USA) before incubation with primary antibody: phospho-ATM (#5883; Cell Signaling Technologies), phospho-CHK2 (#A94672; antibodies.com, Cambridge, UK), CHK2 (#A96222; antibodies.com), phospho-P53 (#9286; Cell Signaling Technologies), P53 (#A95479; antibodies.com), and vimentin (#12826; Cell Signaling Technologies), all at a concentration of 1/1000 overnight at 4 °C. Secondary antibody was rabbit HRP (#P0448; Dako, Glostrup, Denmark) at 1/5000), and HRP was detected using the West Femto Chemiluminescence Detection Kit (#34094; Pierce, ThermoFisher, Loughborough, UK) on the BioRad ChemiDoc MP (Bio-Rad, Watford, UK). Protein concentrations were also assessed on Western blots using Coomassie blue. Band quantification was carried out using Image Lab Software (BioRad, Watford, UK) and normalized to either unphosphorylated protein or Coomassie blue.

### Immune cell analysis from whole blood

Twenty microliters of whole blood was mixed with 20 μL 0.5 M EDTA and stored at 4 °C. On the day of analysis, blood was warmed to room temperature and mixed well. Whole-blood analysis was performed on the Horiba Medical Sci Vet abc Plus (scil animal care company GmbH, Viernheim, Germany). The following information from the analysis report was extracted: platelet cell counts, white and red blood cell counts, and percentage and cell count for lymphocytes, monocytes, granulocytes, and eosinophils and hemoglobin (g/dL).

### Flow cytometry

Spleens were stored in 90% FBS/10% DMSO at −80 °C until the day of staining. On thawing, spleens were homogenized through a 100-μm filter using a syringe plunger, washed, and stained for immune cells. Along with the primary antibody, cells were stained with fc block (#553142; BD, Wokingham, UK) and live dead stain (#L34975; Invitrogen, ThermoFisher, Loughborough, UK) diluted in 1% FBS/PBS for 45 min at 4 °C. Cells were then fixed in 1% PFA/PBS overnight. Before analysis, cells were washed with 1% FBS/PBS and filtered through a 0.45-μM filter.

Isolated cells from the spleen were incubated with the following primary antibodies to lymphoid or myeloid cells:

- Lymphoid panel: CD3Ɛ BV510 (#100353; Biolegend, London, UK; 1/250), CD19 BV605 (#115540; Biolegend, London, UK; 1/100), CD25 PE (#102008; Biolegend, London, UK; 1/400), CD4 PerCP/ Cy5.5 (#100434; Biolegend, London, UK; 1/100), NK1.1 PECy7 (#108714; Biolegend, London, UK; 1/400), CD8 AlexaFluor647 (#100724; Biolegend, London, UK; 1/100), CD45 BV421 (#103134; Biolegend, London, UK; 1/250)
- Myeloid panel: CD11b BV750 (#101267; Biolegend, London, UK; 1/100), F4/80 BV650 (#123149; Biolegend, London, UK; 1/25), Ly6c PE (#128008; Biolegend, London, UK; 1/300), Ly6G PerCP/Cy5.5 (#127616; Biolegend, London, UK; 1/100), MHC II PECy7 (#107630; Biolegend, London, UK; 1/300), CD11c PE 610 (#61-0114-82; Invitrogen, ThermoFisher, Loughborough, UK; 1/100), CD163 APC (#155306; Biolegend, London, UK; 1/400), CD206 AlexaFluor700 (#141734; Biolegend, London, UK; 1/100)

Immune cells were analyzed from 3–6 non–tumor-bearing mice per group by flow cytometry using the Cytek Aurora (Cytek Biosciences, Amsterdam, The Netherlands) following exclusion of debris, doublets, and dead cells. CD45 was used to detect immune cells and individual cell types analyzed using the following markers: B cells: CD45+, CD19+, CD3–, 6; T cells: CD45+, CD19+, CD3–, CD8+/CD4+7; NK cells: CD45+, CD19+, CD3–, NK1.1+8; dendritic cells: CD45+, CD11b+, CD11c+, MHCII+9; neutrophils: CD45+, CD11b+, Ly6G+10; total macrophages: CD45+, CD11b+, Ly6G−/Ly6C low, F480+11; M2-like macrophages: CD45+, CD11b+, Ly6G−/Ly6C low, F480+, CD206+, CD163+ and cells analysed using SpectroFlo, Aurora software. The gating strategy to identify individual immune cell populations for lymphoid and myeloid cells is shown in Figures S1 and S2, respectively.

### Statistical analysis

Statistical analysis was performed using GraphPad PRISM software version 10. Analysis of multiple datasets was performed using 1-way ANOVA, assuming unequal variance, followed by Tukey’s post hoc analysis to determine significance between groups. Data were considered significantly altered if *p* < .05.

## Results

### Effects of adding ATM inhibitor AZD0156 to high-dose Ra-223 on skeletal metastasis and DNA damage repair response proteins in a mouse model of human PC3 prostate cancer bone metastasis

**Figure 1:**
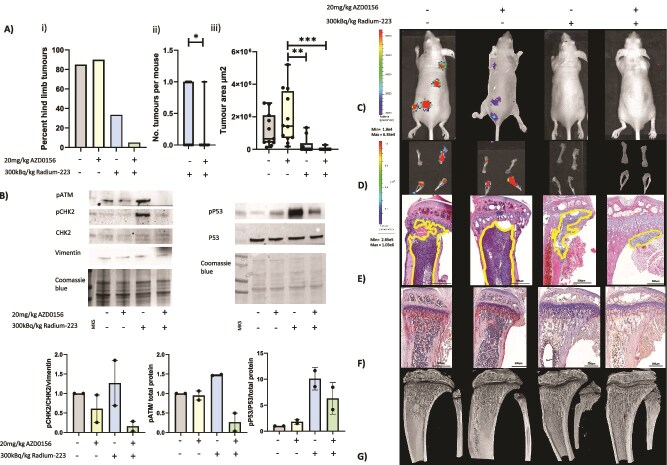
Effects of combining AZD0156 with radium-223 (Ra-223) on PC3 prostate cancer bone metastasis. Six- to 8-wk-old male BALB/c nude mice received an intracardiac injection of PC3-Luc prostate cancer cells, *n* = 10 per group. Seven days after tumor cell injection mice were administered 20 mg/kg/d ATM inhibitor (ATMi) (AZD0156), 300 kBq/kg/wk Ra-223, starting 24 h after the ATMi or a combination of ATMi and Ra-223. Panel A (i) shows the percentage of mice with hind-limb tumors, (ii) shows the number of hind-limb tumors per mouse, (iii) shows tumor size determined histologically. (B) Tumors were isolated from the hind limbs of BALB/c mice following treatment with 300 kBq/kg Ra-223 in combination with 20 mg/kg AZD0156. Representative Western blots showing the effects on the phosphorylation of ATM, CHK2, and p53 are shown in panel B. Panels C and D show photographic IVIS images of tumors in vivo and ex vivo, respectively. Panel E shows H&E-stained histological sections of representative tumor-bearing mice, with the tumor extending beyond the cropped photograph in placebo and AZD0156-alone images. (F) Photomicrographs of TRAP/c-stained histological sections from non–tumor-bearing mice and (G) micro-CT images of trabecular bone taken from non–tumor-bearing tibias from BALB/c nude mice treated with 300 kBq/kg, 20 mg/kg AZD0156 alone, or in combination. One-way ANOVA with Tukey’s post hoc test was used to compare all experimental groups with each other. ^*^p < .05, ^**^p < .01, ^***^p < .005. Panel A shows median with lower and upper quartiles and extremes. Data shown in panel B are means +/− SDs; individual data points are shown.

Initial, proof-of-principle, experiments were carried out using a high dose of Ra-223, 300 kBq/kg, as this has previously been shown to reduce bone metastasis from prostate cancer in mouse models.^27^ Clinically relevant doses of ATMi, administered daily starting 24 h before Ra-223, were chosen to prevent radiation-induced ATM-mediated DNA damage response activation. Using this strategy, administration of ATMi AZD0156 alone had no effect on tumor burden in mouse bone, whereas 300 kBq/kg Ra-223 reduced the number of mice with skeletal metastases by 60.9% compared with placebo. Administration of AZD0156 starting prior to Ra-223 further reduced skeletal metastasis by 94.1% (Figure 1Ai). Importantly, the number of hind limb tumors was reduced by 85.2% in mice treated with AZD0156 starting prior to Ra-223 compared with Ra-223 alone (*p* < .05; Figure 1Aii). Furthermore, histological analysis of bone sections showed an 86.2% reduction in tumor size between AZD0156 alone and a combination of AZD0156 and Ra-223 (*p* < .01) and a 67.7% reduction in volume with Ra-223 combined with AZD0156 and Ra-223 alone (*p* > .05) when administration of the ATMi was started 24 h before the Ra-223 (Figure 1Aii). Taken together, these data suggest that AZD0156 is an effective radiosensitizer for increasing the anti-tumor efficacy of Ra-223 in bone.

CHK2 and TP53 are direct downstream targets of ATM, with phosphorylation at Thr68 and Ser15, respectively, being established markers of cellular ATM kinase activity in response to DNA damage. Autophosphorylation of ATM as Ser1981 also stimulates ATM kinase activity.^28^ To demonstrate the activation of this DNA damage response pathway by Ra-223 and the ability of ATMi to inhibit this, we analyzed phosphorylation of ATM, CHK2, and p53 in PC3 bone tumors isolated from the hind limbs of BALB/c nude mice (Figure 1B). As expected, administration of ATMi alone did not significantly alter ATM, CHK2, or p53 phosphorylation; 300 kBq/kg Ra-223 increased phosphorylation of ATM, CHK2, and p53 by 1.4-fold, 1.3-fold, and 18.2-fold, respectively, compared with placebo; and administration of ATMi starting 24 h before Ra-223 reduced Ra-223–induced phosphorylation of ATM by 5.3-fold, CHK2 by 8-fold, and p53 by 1.8-fold (Figure 1B), confirming that AZD0156 blocks Ra-223–induced activation of the ATM pathway in vivo.

**Figure 2:**
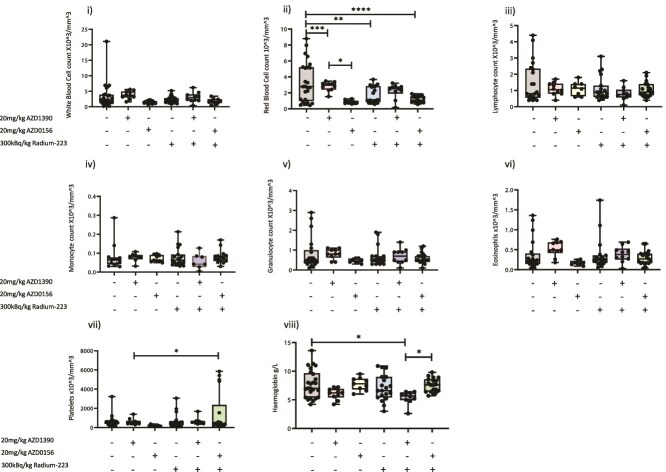
Effects of ATM inhibitors (ATMi)/radium-223 (Ra-223) on immune cell populations in the whole blood of BALB/c nude mice. Six- to 8-wk-old male BALB/c nude mice received an intracardiac injection of PC3-Luc prostate cancer cells. Seven days after tumor cell injection mice were administered 20 mg/kg AZD1390 or 20 mg/kg AZD0156 starting 24 h before Ra-223 and/or 3-weekly doses of 300 kBq/kg or 50 kBq/kg Ra-223 for 18 d. *n* = 10 per group for AZD1390 and AZD0156 alone, 50 kBq/kg Ra-223, AZD1390 with 50 kBq/kg Ra-223, and AZD1390 with 300 kBq/kg Ra-223. *n* = 20 per group for placebo, 300 kBq/kg Ra-223, AZD0156 with 300 kBq/kg Ra-223. Twenty microliters of whole blood was analyzed using SciVet and data shown are mm^3^ for the numbers of white blood cells (i), red blood cells (ii), lymphocytes (iii), monocytes (iv), granulocytes (v), eosinophils (vi), platelets (vii), and amount of hemoglobin in grams per liter (viii). Data shown are medians with lower and upper quartiles and extremes; individual data points are shown. One-way ANOVA with Tukey’s post hoc test was performed: ^*^p < .05, ^**^p < .01, ^***^p < .005, ^****^p < .001.

Radium-223 rapidly binds to bone, emitting alpha particles that affect all cells within 100 μm of its deposition. We therefore investigated the effects of high-dose Ra-223 and ATMi alone or in combination on the bone environment (Figure 1E-G and Table 1) and immune cell populations (Figure 2). To ensure that effects on bone were not influenced by lesions caused by overt metastasis, bone analysis was performed on tibias from mice injected with tumor cells where no overt metastases were detected. Administration of AZD0156 had no effect on trabecular bone volume, trabecular thickness, numbers of osteoclasts, or numbers of osteoblasts compared with placebo. However, AZD0156 reduced the number of closed pores (*p* < .0001). The administration of 300 kBq/kg Ra-223 led to significant increases in trabecular bone volume and trabecular thickness compared with placebo or AZD0156 (*p* < .001 for percentage of bone volume/tissue volume (BV/TV%); *p* < .0001 for trabecular thickness). Radium-223 also reduced numbers of osteoclasts (*p* < .0001; *p* < .0001 compared with placebo or AZD0156, respectively) and the area of bone in contact with osteoclasts, as represented by numbers of osteoclasts per millimeter of bone surface and osteoclast perimeter. Interestingly, neither Ra-223 or AZD0156 had effects on osteoblast number or area of bone in contact with osteoblasts. Combining AZD0156 with Ra-223 had similar effects on bone volume trabecular thickness, pore closure, and osteoclasts in bone as Ra-223 alone, demonstrating that combining these drugs does not increase antiresorptive effects of Ra-223 (Table 1).

**Table 1:** Effects of ATM inhibitors (ATMi) AZD0156 or AZD01390 and radium-223 (Ra-223) alone or in combination on bone turnover and morphology in BALB/c nude mice.

**Treatment** **(BALB/c** **nude mice)**	**%BV/TV**	**Trabecular** **thickness (μm)**	**No. of** **closed pores**	**No. of** **osteoclasts**	**No. of** **osteoclasts per** **bone surface**	**Osteoclast perimeter** **(μm)**	**No. of** **osteoblasts**	**No. of** **osteoblasts** **per bone** **surface**	**Osteoblast** **perimeter** **(μm)**
**Placebo**	10.21 +/− 0.86	0.0330 +/− 0.0016	146.0 +/− 10.65	78.0 +/− 5.98	20.48 +/− 1.7	3172.51 +/− 325.92	81.5 +/− 15.64	14.43 +/− 2.07	2384.57 +/− 402.67
**AZD0156**	11.68 +/− 0.58	0.0373 +/− 0.0013	7.67 +/− 3.71^****^ (vs placebo)	63.33 +/− 6.74	25.61 +/− 0.93	3474.49 +/− 240.37	111.0 +/− 24.43	20.43 +/− 5.62	3096.74 +/− 771.4
**AZD1390**	8.80 +/− 0.76	0.0335 +/− 0.0	79.33 +/− 19.38^*^ (vs placebo)	54.33 +/− 6.36	20.58 +/− 3.0	2592.31 +/− 552.76	37.67 +/− 13.37 *p* ***=*** .058 vs AZD0156	9.89 +/− 4.11	1168.11 +/− 392.85
**50 kBq/kg** **Ra-223**	10.09 +/− 0.78	0.0377 +/− 0.0012	248.0 +/− 60.43	44.29 +/− 6.05^**^ (vs placebo)	15.53 +/− 2.15^***^ (vs placebo)	2059.6 +/− 285.49^*^ (vs placebo)	67.0 +/− 13.06	16.68 +/− 0.76	2312.14 +/− 154.97
**300 kBq/kg** **Ra-223**	17.45 +/− 1.48^***^ (vs placebo, AZD1390, 50 kBq Ra-223)	0.0533 +/− 0.0013^****^ (vs placebo, ATMi, 50 kBq Ra-223)	201.56 +/− 37.15^**^ (vs AZD0156)	34.89 +/− 3.34^****^ (vs placebo)^*^ (vs AZD0156)	8.92 +/− 1.07^****^ (vs placebo, AZD0156)^**^ (vs AZD1390)^*^ (vs 50 kBq Ra-223)	1314.84 +/− 176.11^***^ (vs placebo)^**^ (vs AZD0156)	67.78 +/− 12.15	16.17 +/− 2.47	248 492 +/− 434.23
**AZD0156 +** **300 kBq/kg** **Ra-223**	13.53 +/− 2.06	0.0517 +/− 0.0018^****^ (vs placebo, AZD0156)	105.25 +/− 22.22^*^ (vs AZD0156)	38.25 +/− 8.55^**^ (vs placebo)	7.48 +/− 2.17^***^ (vs placebo)^****^ (vs AZD0156)	1076.99 +/− 304.07^***^ (vs placebo)^**^ (vs AZD0156)	102.0 +/− 18.57	24.25 +/− 3.55	4031.05 +/− 857.89
**AZD1390 +** **300 kBq/kg** **Ra-223**	13.24 +/− 0.76	0.0437 +/− 0.0006^*^ (vs AZD1390, 300 kBq Ra-223)	318.67 +/− 62.03^**^ (vs placebo, AZD1390)	32.67 +/− 1.86^****^ (vs placebo)	10.74 +/− 1.7^*^ (vs placebo, AZD1390)	1268.98 +/− 54.65^**^ (vs placebo)	27.67 +/− 7.45 *p =* .075 vs placebo	8.76 +/− 2.09	1087.72 +/− 275.1
**AZD1390** **+ 50 kBq/kg** **Ra-223**	7.12 +/− 0.28	0.0399 +/− 0.0021^*^ (vs placebo)	104.67 +/− 24.34	59.33 +/− 11.35	18.33 +/− 2.8	2314.93 +/− 481.41	41.67 +/− 6.84	10.38 +/− 2.2	1265.86 +/− 252.84

Tumor-free tibia were taken from male BALB/c nude mice and fixed in 4% PFA after PC3 tumor cell injection following 18 sequential days of 20 mg/kg AZD1390 or 20 mg/kg AZD0156 and or 3-weekly doses of 300 kBq/kg Ra-223. Number of tibias scanned per group: placebo, *n* = 6; AZD1390 and AZD0156 alone, *n* = 3; 300 kBq/kg Ra-223, *n* = 9; AZD1390 with 300 kBq/kg Ra-223, *n* = 3; and AZD0156 with 300 kBq/kg Ra-223, *n* = 4. Data shown are means +/− SEM. One-way ANOVA with Tukey’s post hoc test was performed. ^*^p < .05, ^**^p < .01, ^***^p < .005, ^****^p < .001.

Whole-blood analysis in BALB/c nude mice indicated that AZD0156 caused no detectable effects on numbers of immune cells; however, reduced numbers of red blood cells were found in peripheral blood of AZD0156-treated mice (*p* < .001 compared with placebo). When administered in combination with Ra-223, however, no further reductions in numbers of red blood cells were observed. Interestingly, AZD1390 caused a very small increase in white blood cells compared with control (*p* < .1); however, this ATMi did not affect the red blood cell count, either when administered alone or in combination with Ra-223 where AZD1390 treatment commenced 24 h before Ra-223 (Figure 2).

### Effects of adding AZD1390 to a high or clinically relevant dose of Ra-223 on skeletal metastasis and bone in a mouse model of human PC3 prostate cancer bone metastasis

**Figure 3:**
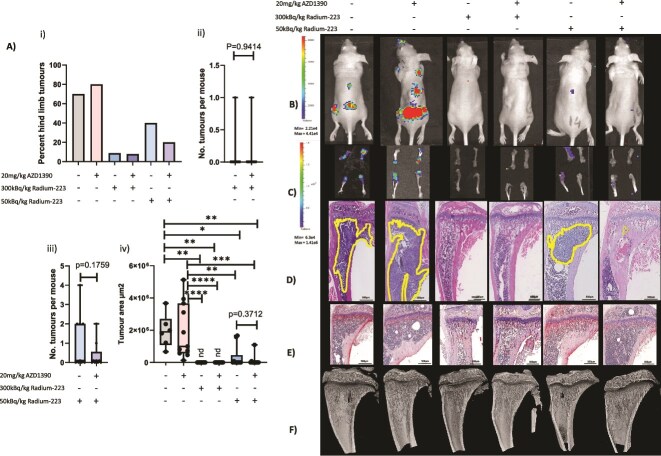
Effects of combining AZD1390 with radium-223 (Ra-223) on PC3 prostate cancer bone metastasis. Six- to 8-wk-old male BALB/c nude mice received an intracardiac injection of PC3-Luc prostate cancer cells, *n* = 10 per group. Seven days after tumor cell injection mice were administered 20 mg/kg/d ATM inhibitor (ATMi) (AZD1390), 300 kBq/kg/wk, or 50 kBq/kg/wk Ra-223 starting 24 h after the ATMi or a combination of ATMi and Ra-223. Panel A (i) shows the percentage of mice with hind-limb tumors, (ii) shows the number of hind-limb tumors in mice treated with 300 kBq/kg/wk Ra-223 alone or in combination with AZD1390, (iii) shows the number of hind-limb tumors in mice treated with 50 kBq/kg/wk Ra-223 alone or in combination with AZD1390, (iv) shows tumor size by immunohistochemistry. (B) Photographic IVIS images of live mice with tumor burden shown in color. (C) Examples of ex vivo hind-limb images taken with the IVIS. (D) H&E-stained histological sections of representative tumor-bearing mice, with the tumor extending beyond cropped photograph in placebo and AZD1390-alone images. (E) Photomicrographs of TRAP/c from non–tumor-bearing mice. (F) Micro-CT images of trabecular bone taken from non–tumor-bearing tibias from BALB/c nude mice treated with 300 kBq/kg or 50 kBq/kg Ra-223, 20 mg/kg AZD1390 alone or in combination. One-way ANOVA with Tukey’s post hoc test compared all experimental groups with each other. ^*^p < .05, ^**^p < .01, ^***^p < .005, ^****^p < .001. Data shown are medians with lower and upper quartiles and extremes; individual data points are shown.

Patients with prostate cancer bone metastases are treated with a significantly lower dose of Ra-223 than the 300 kBq/kg used in data presented in Figure 1 (generally ~55 kBq/kg). Therefore, in order to determine whether ATMi synergistically increases the anti-tumor effects of Ra-223 we reduced the dose given to mice to a more clinically relevant dose of 50 kBq/kg and compared this with 300 kBq/kg Ra-223. Treatment with 300 kBq/kg Ra-223 reduced the number of mice bearing tumors by 87.4% compared with placebo, and the addition of AZD1390 did not alter this result. Treatment with a clinically relevant dose of Ra-223 reduced the number of mice with tumors by 42.9%, and combining AZD1390 with 50 kBq/kg Ra-223 reduced this further to 71.4% (Figure 3Ai). The number of hind limb tumors in mice given 300 kBq/kg Ra-223 was not significantly different when AZD1390 was added (Figure 3Aii), but a 60% reduction in the number of hind limbs with tumor was seen when AZD1390 was added to 50 kBq/kg Ra-223 versus Ra-223 alone (Figure 3Aiii; *p* = .1759) and this was confirmed in histological sections, with no tumor detection in mice treated with 300 kBq/kg Ra-223 with or without AZD1390 and a 47% reduction in tumor size with a combination of AZD1390 and 50 kBq/kg Ra-223 treatment versus Ra-223 alone (Figure 3Aiv; *p* = .3712), further suggesting radiosensitizing effects of AZD1390 in the bone environment.

A combination of 300 kBq/kg Ra-223 with AZD1390 also led to increases in trabecular bone volume and trabecular thickness versus placebo or AZD1390 (*p* < .001 for BV/TV%, *p* < .0001 for trabecular thickness), whereas 50 kBq/kg Ra-223 had significantly less pronounced effects on bone than 300 kBq/kg; 50 kBq/kg Ra-223 had no effect on trabecular bone volume or trabecular thickness when given alone, but increased trabecular thickness when given in combination with AZD1390 versus placebo (*p* < .05), with AZD1390 alone increasing the number of closed pores versus placebo (*p* < .01). Numbers of osteoclasts were not reduced in 50 kBq/kg Ra-223 versus AZD1390, although 300 kBq/kg Ra-223 alone did reduce numbers of osteoclasts (*p* < .01), numbers of osteoclasts per bone surface (*p* < .011), and osteoclast perimeter (*p* < .05) compared with placebo. However, these reductions in osteoclasts were not observed when 50 kBq/kg Ra-223 was administered in combination with AZD1390 (Figure 3D–F, Table 1).

**Figure 4:**
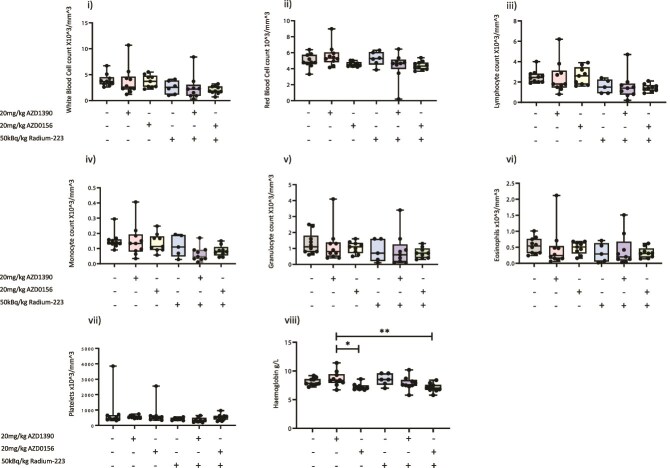
Effects of ATM inhibitors (ATMi)/radium-223 (Ra-223) on C57BL/6 whole blood. Six- to 8-wk-old male C57BL/6 mice received an intracardiac injection of RM1-Luc prostate cancer cells. Two days after tumor cell injection mice were administered 20 mg/kg/d ATMi (AZD1390), 50 kBq/kg/wk Ra-223, or ATMi treatment starting 24 h before Ra-223, and mice were culled 14 d after initial tumor cell injection (*n* = 10/group). Twenty microliters of whole blood was stored in 20 μL of 0.5-M EDTA at 4 °C prior to analysis. SciVet analysis was used to determine the number of white blood cells/mL (i), red blood cells/mL (ii), lymphocytes/mL (iii), monocytes/mL (iv), granulocytes/mL (v), eosinophils/mL (vi), platelets/mL (vii), and amount of hemoglobin in grams per liter (viii). One-way ANOVA with Tukey’s post hoc test was performed. Data shown are medians with lower and upper quartiles and extremes. ^*^p < .05, ^**^p < .01.

Importantly, in addition to showing promising anti-tumor effects, combining AZD1390 with Ra-223 did not adversely affect red or white blood cell or platelet counts in peripheral blood (Figure 4), suggesting that this combination may be suitable for taking forward into clinical trials. Administration of AZD1390, Ra-223 alone, or a combination of Ra-223 and AZD1390 did not alter numbers of cells within the peripheral circulation. These data suggest that AZD1390 may be the least hemotoxic compound tested and therefore would be a better option for combining with Ra-223 for further analysis.

### Effects of combining ATMi with a clinically relevant dose of Ra-223 on early- and late-stage skeletal metastasis in a C57BL/6 mouse model of prostate cancer bone metastasis

Because high-dose Ra-223 had a profound effect on PC3 skeletal tumors in our mouse model and Ra-223 is used at a lower dose in humans, experiments were repeated in a second, immune-competent model, using a therapeutically relevant dose of 50 kBq/kg Ra-223. In addition, we focused on the ATMi AZD1390, as this drug appears to be less hemotoxic than AZD0156 (Figure 2).

**Figure 5:**
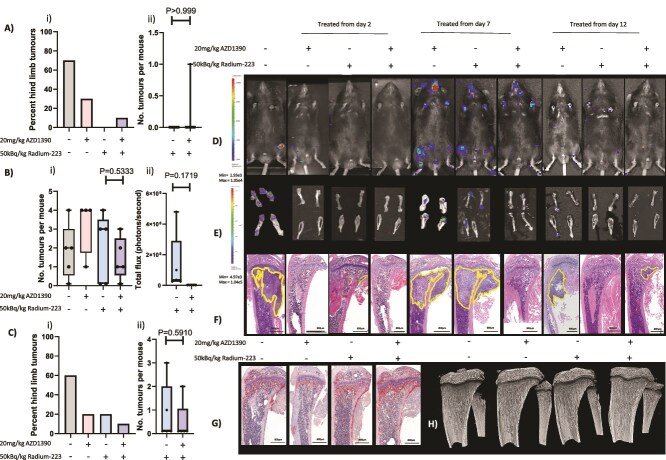
Effects of combining AZD1390 with radium-223 (Ra-223) on RM1 prostate cancer bone metastasis. Six- to 8-wk-old male C57BL/6 mice received an intracardiac injection of RM1-Luc prostate cancer cells. (A) Two days after tumor injection, *n* = 10 per group; (B) 7 days after tumor injection, *n* = 5 per group; or (C) 12 days after tumor injection, *n* = 5 per group, mice were administered 20 mg/kg/day ATM inhibitor (ATMi) (AZD1390), or 50 kBq/kg/wk Ra-223, or a combination of ATMi and Ra-223, with Ra-223 treatment starting 24 h after the ATMi. (A/Ci) Percentage of mice with hind-limb tumors from 2 d and 12 d after the start of treatment post–tumor cell injection, respectively. (A/Cii) The number of hind-limb tumors in mice treated with 50 kBq/kg/wk Ra-223 alone or in combination with AZD1390 from 2 d and 12 d after the start of treatment post–tumor cell injection, respectively. (Bi) The number of hind-limb tumors in mice from 7 d after the start of treatment post–tumor cell injection. (Bii) IVIS imaging photons per second in ex vivo hind limbs from mice treated with 50 kBq/kg/wk Ra-223 alone or in combination with AZD1390 from 7 d after the start of treatment post–tumor cell injection. (D) Photographic IVIS images of live mice with tumor burden shown in color. (E) Examples of ex vivo hind-limb images taken with the IVIS. (F) H&E-stained histological sections of representative tumor-bearing mice. (G) Photomicrographs of TRAP/c-stained histological sections of trabecular bone taken from non–tumor-bearing tibias from C57BL/6 mice treated with 50 kBq/kg Ra-223 or 20 mg/kg AZD1390 alone or in combination with treatment starting at day 2. (H) Micro-CT images of non–tumor-bearing tibias from C57BL/6 mice treated with 50 kBq/kg Ra-223 or 20 mg/kg AZD1390 alone or in combination with treatment starting at day 2. Statistical analysis was performed using 1-way ANOVA with Tukey’s post hoc test comparing all experimental groups with each other. Data shown are medians with lower and upper quartiles and extremes; individual data points are shown.

Using this model, we also assessed the efficacy of combining AZD1390 with 50 kBq/kg Ra-223 against early- and late-stage prostate cancer to determine if this combination is useful for the treatment of large as well as small tumors. Figure 5Ai and 5F and Figure 3Aii demonstrate that treating early (day 2) with Ra-223 eliminated RM1 tumor cells (to levels below methods of detection) and the addition of AZD1390 to a clinical dose of Ra-223 did not alter these results (*p* > .999).

Interestingly, when treatment was delayed simulating late-stage disease, starting treatment 12 d after tumor injection (Figure 5Ci), both Ra-223 and AZD1390 treatment reduced bone metastases by 66.6% compared with placebo and combining AZD1390 with Ra-223 reduced this further to 83.3.%. The number of hind-limb tumors in these mice was reduced by 50% with the combination of AZD1390 and Ra-223 versus Ra-223 alone, demonstrating the potential benefit of radiosensitization with ATMi prior to, Ra-223 even at late-stage prostate cancer bone metastasis. The mice in this late-stage treatment experiment were culled 2 d post–Ra-223 treatment and therefore tumors in these mice had not become resistant to treatment. Mice treated 7 d post–tumor injection showed tumor resistance, as the numbers of hind-limb tumors per mouse were not significantly different between treatments (Figure 5Bi); however, interestingly, tumor size measured by ex vivo imaging of hind limbs utilising an in vivo imaging system (IVIS) showed a 99.3% reduction in tumor size measured in photons per second in combined AZD1390 and Ra-223 treatment versus Ra-223 alone (Figure 5Bii; *p* = .1719), and tumors in the bones of mice treated with a combination of AZD1390 and Ra-223, where AZD1390 was started 24 h before Ra-223 were not detectable by immunohistochemistry (Figure 5F).

A dose of 50 kBq/kg Ra-223 resulted in a small, nonsignificant increase in trabecular bone volume and had no effect on trabecular thickness when given alone; furthermore, the addition of AZD1390 did not alter bone volume in C57BL/6 mice (Table 2, Figure 5H). A dose of 50 kBq/kg Ra-223 alone increased the number of closed pores in trabecular bone by 187% compared with placebo (*p* = .0276), but this lost significance when given in combination with AZD1390: a 166% increase with AZD1390 compared with placebo (*p* > .05).

**Table 2:** Effects of ATM inhibitors (ATMi) AZD0156 or AZD01390 and radium-223 (Ra-223) alone or in combination on bone turnover and morphology in C57BL/6 mice.

**Treatment** **(C57BL/6)**	**%BV/TV**	**Trabecular** **thickness (μm)**	**No. of** **closed pores**	**No. of** **osteoclasts**	**No. of** **osteoclasts** **per bone** **surface**	**Osteoclast** **perimeter (μm)**	**No. of** **osteoblasts**	**No. of** **osteoblasts** **per bone** **surface**	**Osteoblast** **perimeter (μm)**
**Placebo**	12.12 +/− 0.92	0.0280 +/− 0.0011	541.6 +/− 80.12	86.2 +/− 6.4	4.29 +/− 0.32	4243.46 +/− 452.47	120 +/− 31.72	5.61 +/− 1.13	3161.20 +/− 907.62
**AZD1390**	13.54 +/− 0.68	0.0290 +/− 0.0005	743.4 +/− 91.08	108.2 +/− 8.9	5.72 +/− 0.45	3930.37 +/− 289.84	131.17 +/− 55.99	6.55 +/− 2.48	2002.103 +/− 416.62
**50 kBq/kg** **Ra-223**	14.60 +/− 1.18	0.0289 +/− 0.0006	1012.4 +/− 90.63^*^ (vs placebo)^**^ (vs AZD0156)	92.3 +/− 13.6	5.48 +/− 0.74	3120.06 +/− 630.16	85.33 +/− 12.92	5.19 +/− 0.90	2197.67 +/− 312.19
**AZD1390** **+ 50 kBq/kg** **Ra-223**	13.84 +/− 1.12	0.0303 +/− 0.0009	899.6 +/− 133.41	93.8 +/− 7.2	4.88 +/− 0.31	4430.07 +/− 513.51	140.83 +/− 24.23	7.06 +/− 0.88	3147.93 +/− 272.69

Tumor-free tibia were taken from male C57BL/6 mice and fixed in 4% PFA after RM1 tumor cell injection following 12 sequential days of 20 mg/kg AZD1390 and or 2-weekly doses of 50 kBq/kg Ra-223. *n* = 10 per group. Data shown are means +/− SEM. One-way ANOVA with Tukey’s post hoc test was performed. ^*^p < .05, ^**^p < .01.

Radium-223 did not significantly reduce numbers of osteoclasts, numbers of osteoclasts per bone surface, or osteoclast perimeter (*p* > .05) compared with placebo, alone or in combination with AZD1390 (Figure 5G). A dose of 50 kBq/kg alone reduced osteoblast number by 29% (*p* = .7897), but this effect was reversed with the addition of AZD1390.

### Effects of combining ATMi with a clinically relevant dose of Ra-223 on systemic immune cell populations

**Figure 6:**
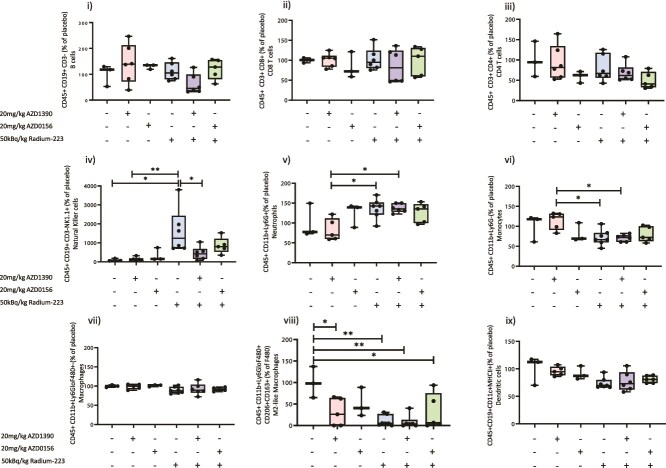
Effects of ATM inhibitors (ATMi)/radium-223 (Ra-223) on systemic immune cells. Spleens from C57BL/6 mice were dissected from mice 14 days after RM1 tumor cell injection following 12 sequential days of 20 mg/kg AZD1390 or 20 mg/kg AZD0156 and/or 2-weekly doses of 50 kBq/kg Ra-223. Placebo- and AZD0156-treated mice, *n* = 3 per group; combination AZD0156 and Ra-223, *n* = 5; and all other groups, *n* = 6 per group. Immune cells were stained for flow cytometric analysis and individual cell types assessed using the following criteria: B cells (i): CD45+, CD19+, CD3–; T cells (ii CD8+, iii CD4+): CD45+, CD19+, CD3–, CD8+/CD4+; NK cells (iv): CD45+, CD19+, CD3–, NK1.1+; neutrophils (v): CD45+, CD11b+, Ly6G+; monocytes (vi): CD45+, CD11b+, Ly6G-; total macrophages (vii): CD45+, CD11b+, Ly6G−/ Ly6C low, F480+; M2-like macrophage (viii): CD45+, CD11b+, Ly6G−/Ly6C low, F480+, CD206+, CD163+; dendritic cells (ix): CD45+, CD11b+, CD11c+; MHCII+. Statistical analysis was performed by 1-way ANOVA with Tukey’s post hoc test comparing all experimental groups. Data shown are medians with lower and upper quartiles and extremes. ^*^p < .05, ^**^p < .01.

To facilitate the growth of human prostate cancer cells for studying bone metastasis we used BALB/c nude mice; these mice do not have a thymus and therefore cannot be used to accurately measure effects on immune cells. Because multiple clinical trials with DNA repair inhibitors have recently been stopped due to patients experiencing severe neutropenia^29^ we used a syngeneic mouse model of RM1 prostate cancer bone metastasis to assess the effects of combining ATMi with Ra-223 on immunity to check for possible adverse immune-related effects. Results from Figure 6 show immune cell populations from excised spleens. No significant differences were detected between placebo and ATMi, Ra-223, or combination treatments in CD45+, CD19+, CD3– B cells; CD45+, CD8+ CD8 T cells; CD45+, CD11b+, Ly6G+ neutrophils; CD45+, CD11b+, Ly6G– monocytes; CD45+, CD11b+, Ly6G low, F480+ macrophages; CD45+, CD19+, CD11c+, MHCII+ dendritic cells; or CD45+, CD4+ T cells (Figure 6). However, Ra-223 alone also increased CD45+, CD19+, CD3-, NK1.1+ NK cells number (Figure 6iv) by 1615% compared to placebo (*p* = .0218); interestingly, the addition of AZD0156 to Ra-223 did not significantly alter NK cell number compared with Ra-223 alone, whereas adding AZD1390 to Ra-223 decreased NK cells by 280% compared with Ra-223 alone (*p* = .0344). Ly6G+ neutrophils (Figure 6V) were significantly increased in AZD1390 alone compared with Ra-223 alone by 165% (*p* = .0199) and compared with a combination of AZD1390 and Ra-223 by 166% (*p* = .0213). In contrast, Ly6G– monocytes (Figure 6vi) were reduced with Ra-223 alone by 36.8% (*p* = .0199) and with a combination of AZD1390 and Ra-223 by 37.7% (*p* = .0213) compared with AZD1390, respectively. Analysis of the M2-like (pro-tumor) macrophages showed that ATMi and Ra-223 treatment reduced this subset of cells without altering the total macrophage population (Figures 6vii). ATMi (AZD1390) alone reduced M2-like macrophages by 69.06% (*p* = .028) and ATMi AZD0156 by 49.13% (*p* = .3011), Ra-223 alone by 89.31% (*p* = .0015), and a combination with Ra-223 and ATMi by 67.75% (*p* = .0323) and 91.35% (*p* = .0016) with AZD0156 and AZD1390, respectively. These data suggest that, in addition to radiation-induced tumor cell killing, Ra-223 may induce additional anti-tumor effects by increasing NK 1.1 cells and reducing pro-tumor macrophages. Administration of ATMi prior to Ra-223 may reduce the ability of Ra-223 to stimulate NK 1.1 cells, especially AZD1390, but does not affect Ra-223-induced inhibition of pro-tumor macrophages.

## Discussion

A dose of 55 kBq/kg Ra-223 every 28 d results in improved overall survival and reduced symptomatic skeletal events in patients with symptomatic metastatic CRPC.^30^ Men assigned to have Ra-223 treatment benefit from an increase in life expectancy of approximately 6 mo compared with placebo-treated controls^7^; however, this treatment is not curative. Following exposure to radiation, surviving cells upregulate DNA repair pathways in order to facilitate survival.^28^ Once upregulation of these pathways occurs, tumor cells become resistant to radiation exposure.^31^ Because Ra-223 is an alpha-emitter that is incorporated into areas of bone undergoing active remodeling, this treatment is highly efficient at killing tumor cells that are located in close proximity to the bone cortex (<100 μm).^6^ However, we hypothesize that those tumors located further away from the bone surface receive a sublethal dose, enabling surviving cells to develop resistance mechanisms and subsequently cause relapse. In accordance with this hypothesis, our data show that administration of high doses (300 kBq/kg) of Ra-223 or more clinically relevant doses (50 kBq/kg) both significantly reduce tumor growth in mouse bone, with no significant differences in metastatic burden being observed between the 2 treatment doses, suggesting that 50 kBq/kg is sufficient to kill tumor cells on the bone surface and suggests that increasing the dose may not alter penetrance of alpha particles into tumor cells, resulting in no additional anti-tumor benefits being seen when mice are treated with a higher dose. Interestingly, both the PC3 and RM1 prostate cancer cell lines used in this current study form lytic bone lesions following intracardiac injection. Radium-223 is a calcium mimetic that binds areas of active bone formation; as such, this radiopharmaceutical is currently used to treat osteoblastic disease.^32^ Although early clinical trials in lytic diseases, such as breast cancer bone metastasis, have not demonstrated benefit of adding Ra-223 to standard of care,^33^ our current data suggest that Ra-223 does integrate into bone in lytic metastases and therefore, under optimal circumstances (eg, following combination with an ATMi), Ra-223 may also be useful as a treatment for lytic bone metastasis.

In accordance with previously published data showing that alpha particles activate ATM,^13–15^ Ra-223 increased ATM signaling in prostate cancer bone metastases (Figure 1B). The addition of ATMi to mice receiving Ra-223 reduced radiation-induced phosphorylation of ATM, CHK2, and P53 compared with control or either treatment alone. Given the pivotal role of ATM in activating the DNA damage response and repair pathways, it is likely that the anti-tumor effects observed when ATMi is combined with Ra-223 in prostate cancer bone metastases prevent efficient repair of the DNA strand breaks, leading to tumor cell death; however, before conclusions can be made, this requires further investigation.

Doses of 300 kBq/kg or 50 kBq/kg Ra-223 were highly efficient at reducing bone metastasis. Combining ATMi treatment with 300 kBq/kg Ra-223 reduced metastases to the extent that tumors were only present in 5% of bones out of a total of 10 BALB/c mice treated with AZD0156 and present in 7.9% in BALB/c mouse bone following AZD1390 treatment. In BALB/c mice, administration of the lower, clinically relevant dose of 50 kBq/kg Ra-223 (Figure 3) was as effective at reducing bone metastases as 300 kBq/kg (Figure 1 and Figure 3); however, if an even lower dose results in the same therapeutic benefit while inducing even fewer potential adverse effects remains to be established. Taken together, these data suggest that using lower doses of Ra-223 may be an appropriate approach to reducing tumor growth in bone. Adding AZD1390 to 50 kBq/kg Ra-223 further reduced both the number of tumors and tumor size. It is interesting to note that the location of some tumors was altered after Ra-223 treatment, and on histological analysis none of the tumors in the combination treatment group were located within the bone marrow cavity, suggesting that combination therapy may be more effective at reducing prostate cancer progression in bone than indicated by the IVIS analysis.

In our immune-competent model, 50 kBq/kg Ra-223 eliminated tumor cells when given at an early time point (ie, micro-metastases), and we found that the addition of ATMi had no benefit at this early time point. An additional benefit of adding ATMi to Ra-223 was observed when treating overt metastases in both size and number of hind-limb tumors and this effect may be due to the increase in NK cells and decreased pro-tumor macrophages. It is also probable that benefits of adding ATMi to Ra-223 could only be observed in larger tumors, as Ra-223 alone is extremely effective at killing small tumors that are located in close proximity to the bone surface, preventing any increases in anti-tumor effects from being visible.

In the clinic, Ra-223 is offered to patients with prostate cancer who have late-stage bone disease, when tumors are visible radiologically and/or on PET/MRI.^8^ In these patients Ra-223 has been shown to reduce tumor burden and increase bone mass. Although Ra-223 has minimal side effects, because the hematopoietic stem cell niche resides on the bone surface, this treatment can lead to anemia and neutropenia.^30^ Furthermore, clinical trials using ATMi have been aborted due to significant neutropenic effects.^30^ In accordance with clinical data, our current mouse modeling studies demonstrated increased trabecular bone volume following 300 kBq/kg Ra-223, which was associated with reduced numbers of osteoclasts. However, no changes in tibial bone volume were observed in mice treated with 50 kBq/kg Ra-223 compared with controls despite significant reductions in numbers of osteoclast cells. Neither 300 kBq/kg nor 50 kBq/kg Ra-223 exerted significant effects on numbers of osteoclasts, indicating that the more clinically relevant dose of 50 kBq/kg Ra-223 is the best option to take forward, reducing bone metastasis while minimizing adverse effects on bone homeostasis. In our study, neither AZD0156 nor AZD1390 had any detectable effects on trabecular bone volume, osteoclasts, or osteoblasts and combining these drugs with Ra-223 did not adversely affect bone turnover compared with Ra-223 alone. It is therefore likely that the addition of an ATMi to Ra-223 in a patient population will not increase bone-associated complications.

In agreement with clinical trials data, either AZD0156^30^ or Ra-223 alone^30^ or in combination led to reduced red blood cell counts in immunocompromised mice (Figure 2ii); however, these adverse effects were not seen in immune-competent mice (Figure 4ii). It must be noted that, for studies utilizing immune-competent mice, fewer treatments with Ra-223 and ATMi were given compared with immune-compromised mice due to RM1 tumors developing more rapidly than PC3 tumors in bone. Therefore, it is likely that the longer mice are exposed to Ra-223 with or without ATMi, the more cytotoxic effects build up. Importantly, adding ATMi to Ra-223 did not increase adverse effects on red blood cells, which were observed following treatment with either drug alone.

Interestingly, adverse immune-related effects, presenting as neutropenia, resulted in clinical trials with AZD0156 being terminated^29^ and AstraZeneca removing this drug from future development. In our immune-competent mouse models we did not observe adverse effects on the white blood cell population. Instead, we observed reduced CD4+ cells following AZD0156, and these cells were further reduced with the addition of Ra-223 (Figure 6iii). Furthermore, percentages of NK cells were increased following Ra-223 and these remained high when mice were pretreated with AZD0156. Increased NK cell function and reduced CD4+ cells leading to increased cytotoxic T-cell function have been postulated as one of the mechanisms by which Ra-223 kills tumor cells, beyond the reach of the alpha-radiation.^34^ Although it would be interesting to explore this hypothesis in more detail, because the development of AZD0156 has been halted we did not carry out further investigations into the potential synergy this drug may have when combined with Ra-223. It should also be noted that it is important to investigate the effects of drug treatments on systemic immunity, as presented here. Identifying effects in anti-tumor immunity would require spatial and immunological cell-typing analysis of bone/bone marrow with or without tumor. Due to the complexities of performing downstream analysis in radioactive bone, these types of data have not been generated in the current project.

The ATMi AZD1390 has the ability to cross the blood–brain barrier; therefore, any benefit of combining this drug with Ra-223 against bone metastasis would suggest that combining AZD1390 with other high-LET alpha particles may be of benefit for patient with tumors at other sites, including brain^35^^–^^37^. Our data show that combining AZD1390 with Ra-223 was better at reducing metastatic burden from large (overt metastases) compared with small (micro-metastases) in mouse bone and, in some mice, these metastases became undetectable. Taken together, our data strongly suggest that combining Ra-223 with an ATMi may improve the therapeutic efficacy of Ra-223 alone against bone metastasis, laying the foundation for a future clinical trial testing the efficacy of this combination in a patient population.

## Supplementary Material

Supplementary_figure_1_ziaf129

Supplementary_figure_2_ziaf129

## Data Availability

Data will be made freely available to researchers upon publication of this manuscript and can be accessed by contacting Professor Penelope Ottewell via email: p.d.ottewell@sheffield.ac.uk.
