# Supplementary figures and images for: ATM inhibition increases the anti-tumor efficacy of radium-223 (Ra-223) against prostate cancer bone metastasis in preclinical models
Source: JBMR Plus. 2025 Aug 5;9(10):ziaf129. doi: 10.1093/jbmrpl/ziaf129 (PMC12448381; doi:10.1093/jbmrpl/ziaf129)

**Lymphoid panel**


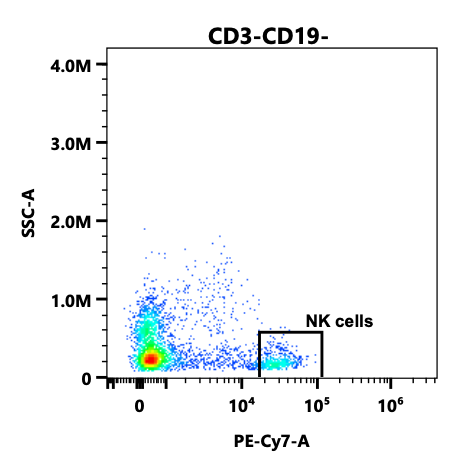

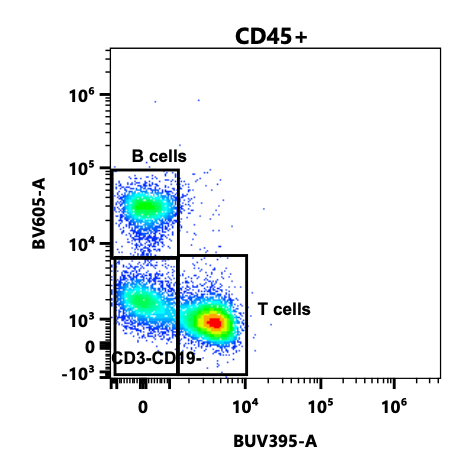

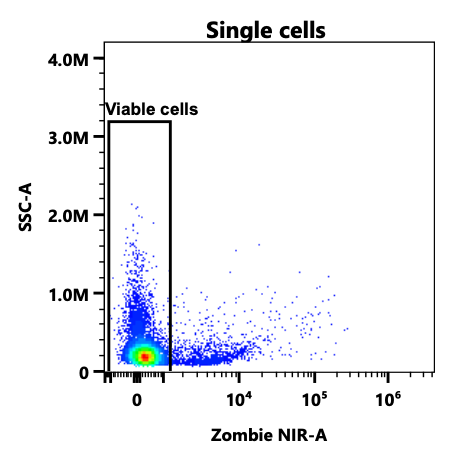

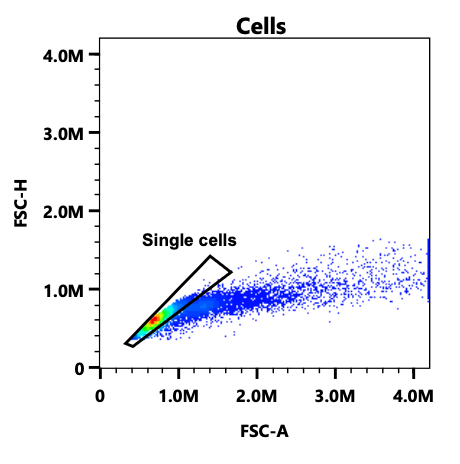

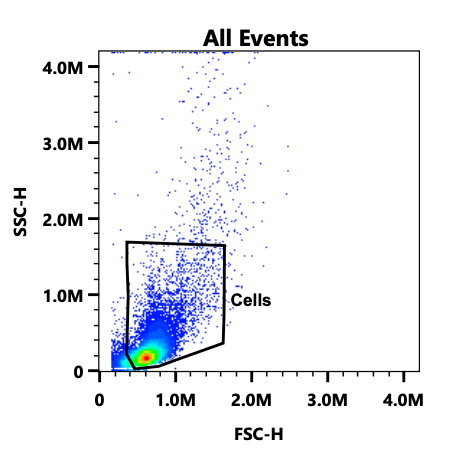


**NK1.1**

**CD19**

**CD3**


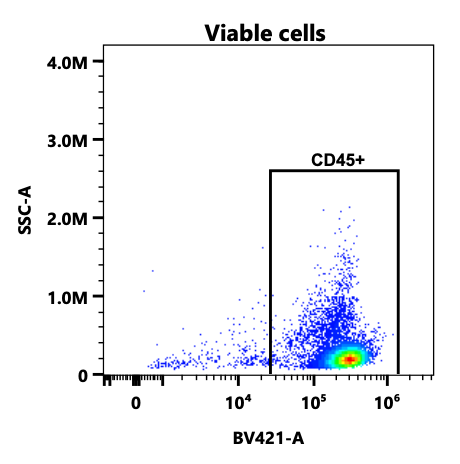


**CD45**


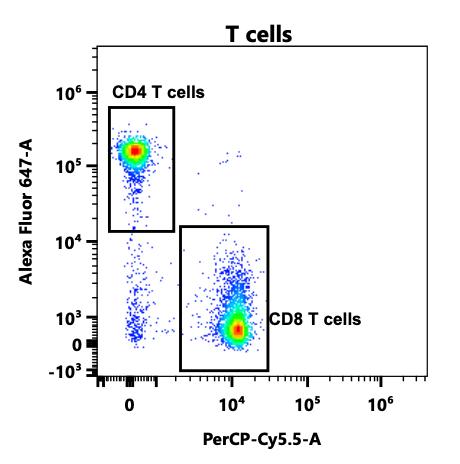


**CD4**

**CD8**

Supplement: Supplementary_figure_1_ziaf129 [file supplementary_figure_1_ziaf129.docx]

**Myeloid panel**


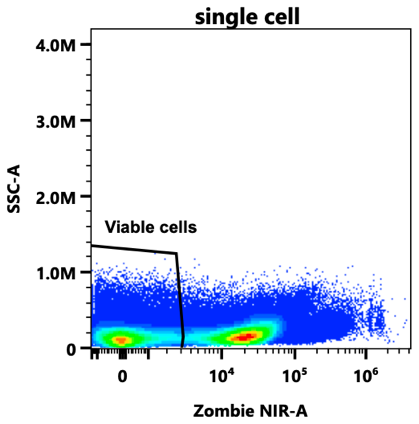


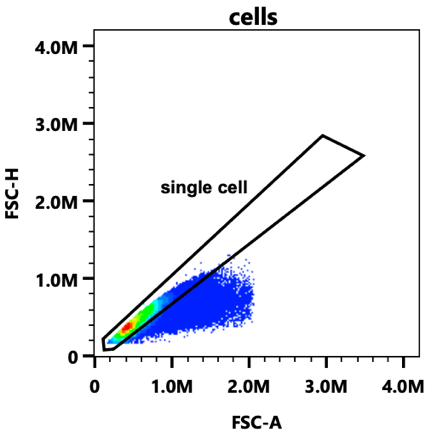

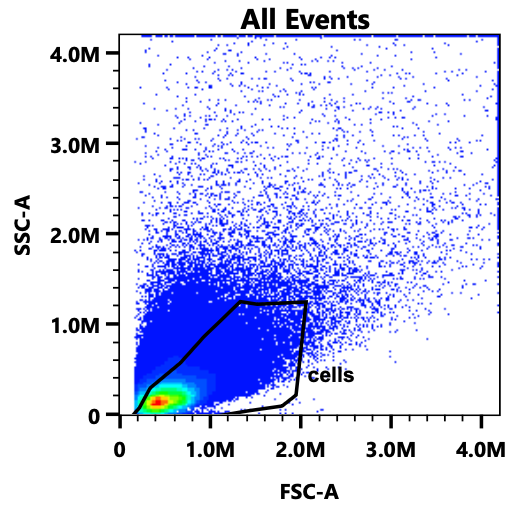


**CD11b**


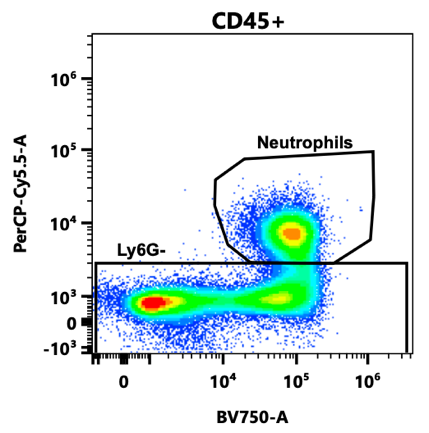


**CD11b**

**Ly6G**


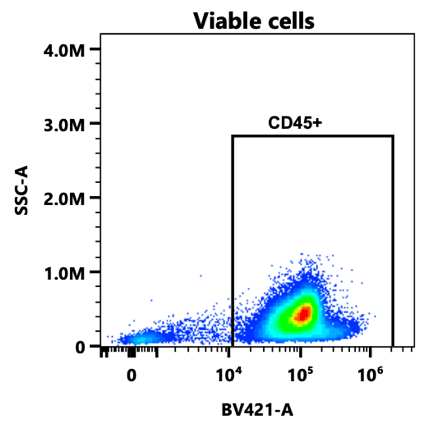


**CD45**


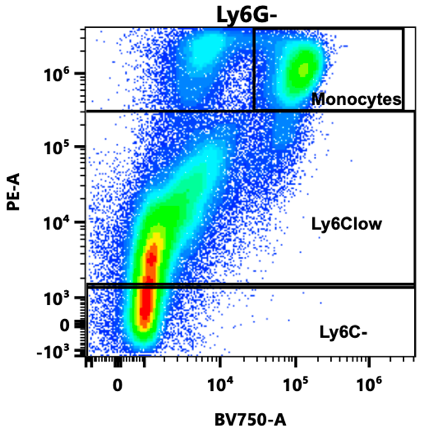


**Ly6C**

**CD11b**


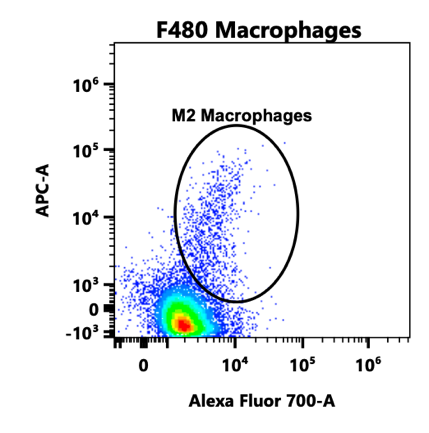


**CD163**

**CD206**


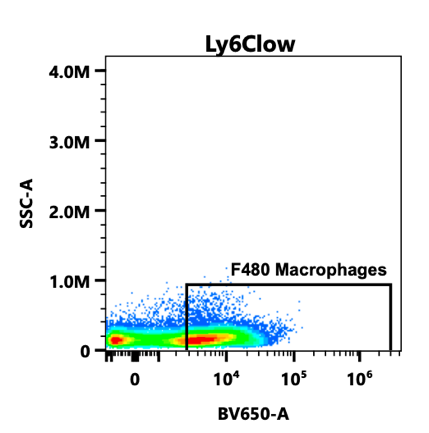


**F4/80**


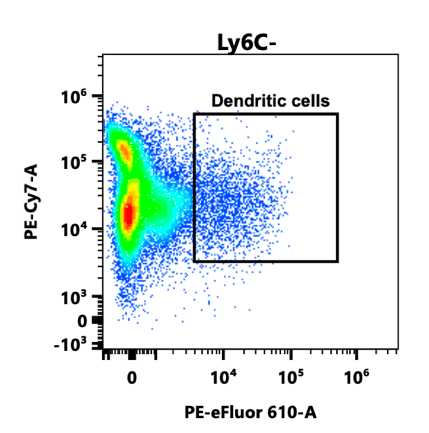


**CD11c**

**MHC II**

Supplement: Supplementary_figure_2_ziaf129 [file supplementary_figure_2_ziaf129.docx]
